# EBNA1: Oncogenic Activity, Immune Evasion and Biochemical Functions Provide Targets for Novel Therapeutic Strategies against Epstein-Barr Virus- Associated Cancers

**DOI:** 10.3390/cancers10040109

**Published:** 2018-04-06

**Authors:** Joanna B. Wilson, Evelyne Manet, Henri Gruffat, Pierre Busson, Marc Blondel, Robin Fahraeus

**Affiliations:** 1 School of Life Sciences, College of Medical, Veterinary and Life Sciences, University of Glasgow, Glasgow G12 8QQ, UK; joanna.wilson@glasgow.ac.uk; 2 CIRI, Centre International de Recherche en Infectiologie, Team Oncogenic Herpesviruses, Inserm, Université Claude Bernard Lyon 1, CNRS, ENS-Lyon, Université de Lyon, F-69007 Lyon, France; evelyne.manet@ens-lyon.fr (E.M.); henri.gruffat@ens-lyon.fr (H.G.); 3 CNRS, UMR 8126, Gustave Roussy, Université Paris Sud, Université Paris-Saclay, F-94805 Villejuif, France; pierre.busson@gustaveroussy.fr; 4 UMR1078 “Génétique, Génomique Fonctionnelle et Biotechnologies”, Inserm, Université de Brest, EFS, IBSAM, CHU, F-29200 Brest, France; marc.blondel@univ-brest.fr; 5 Inserm UMR1162, Université Paris 7, Institut de Génétique Moléculaire, 27 rue Juliette Dodu, F-75010 Paris, France

**Keywords:** EBV, EBNA1, cancer, drug development

## Abstract

The presence of the Epstein-Barr virus (EBV)-encoded nuclear antigen-1 (EBNA1) protein in all EBV-carrying tumours constitutes a marker that distinguishes the virus-associated cancer cells from normal cells and thereby offers opportunities for targeted therapeutic intervention. EBNA1 is essential for viral genome maintenance and also for controlling viral gene expression and without EBNA1, the virus cannot persist. EBNA1 itself has been linked to cell transformation but the underlying mechanism of its oncogenic activity has been unclear. However, recent data are starting to shed light on its growth-promoting pathways, suggesting that targeting EBNA1 can have a direct growth suppressing effect. In order to carry out its tasks, EBNA1 interacts with cellular factors and these interactions are potential therapeutic targets, where the aim would be to cripple the virus and thereby rid the tumour cells of any oncogenic activity related to the virus. Another strategy to target EBNA1 is to interfere with its expression. Controlling the rate of EBNA1 synthesis is critical for the virus to maintain a sufficient level to support viral functions, while at the same time, restricting expression is equally important to prevent the immune system from detecting and destroying EBNA1-positive cells. To achieve this balance EBNA1 has evolved a unique repeat sequence of glycines and alanines that controls its own rate of mRNA translation. As the underlying molecular mechanisms for how this repeat suppresses its own rate of synthesis in *cis* are starting to be better understood, new therapeutic strategies are emerging that aim to modulate the translation of the EBNA1 mRNA. If translation is induced, it could increase the amount of EBNA1-derived antigenic peptides that are presented to the major histocompatibility (MHC) class I pathway and thus, make EBV-carrying cancers better targets for the immune system. If translation is further suppressed, this would provide another means to cripple the virus.

## 1. Introduction

Epstein-Barr virus (EBV) nuclear antigen-1 (EBNA1) is an essential viral protein and is expressed in all EBV-associated tumours as well as all latency programs of the virus, except perhaps for latency 0. It exerts essential functions in viral DNA replication and the episomal genome maintenance, achieved through sequence-specific DNA binding. EBNA1 also interacts with viral and cellular promoters and thereby contributes to the transcriptional regulation of both viral and cellular genes. More recent works shed new light on how this pleiotropic protein exerts its functions and on its oncogenic activity as well as how it regulates its own synthesis in order to evade recognition of the host cells by the immune system. This review is aimed at illustrating how these different insights open new therapeutic approaches aimed at specifically targeting EBV-associated cancers.

### 1.1. EBNA1 Status in EBV-Associated Malignancies

The suspicion that EBNA1 is involved in the oncogenesis of most, if not all, EBV-related malignancies goes back to very early stages of EBV research. The identification of a family of viral antigens consistently detected in the nuclei of malignant cells was reported in the early seventies by Reedman and Klein [1,2,3]. This was made possible by use of a three-layer assay combining natural antibodies from EBV seropositive individuals, complement proteins from an EBV-negative donor and anti-complement fluorescent antibodies. Although this assay was sometimes prone to non-specific cross-reactivity, its implementation was a major breakthrough in the field of EBV research. It gave grounds to the notion that there was a latent EBV infection in various types of proliferating cells. This concept had emerged a few years earlier, with the first report on the detection of EBV DNA by in situ hybridization in tissue sections of Burkitt’s lymphoma (BL) and nasopharyngeal carcinoma (NPC); notably, these are EBV-related tumours characterized by the absence of viral particles and the absence or quasi-absence of lytic products in situ [4]. Subsequently, thanks to the systematic analysis of viral c-DNAs and development of specific protein reagents, it was possible to characterize each protein comprising the EBNA family, particularly EBNA1 [5,6]. In contrast to the other EBNAs, EBNA1 soon appeared to be consistently expressed, in vitro as well as in vivo, in all types of cells subjected to latent EBV infection. Specifically, EBNA1 was detected in the context of natural EBV-related tumours of lymphoid and epithelial origin, as well as in the context of lymphoblastoid cell lines (LCLs) [5,7]. 

This consistency of EBNA1 expression is in accord with the fact that, in all types of latent EBV infection, the viral genome is retained in the form of an extra-chromosomal episome [8,9]. The instances of latently EBV-infected cells where all copies of the viral genome are integrated into cellular chromosomes are rare [10]. It seems that even when some genome copies are integrated, the presence of additional episomal copies is virtually requisite [11,12]. The EBNA1 protein is required to ensure the extra-chromosomal replication of the viral episomes and their balanced segregation at each cell division and therefore their maintenance in an expanding cell population [13,14,15]. Overall, EBNA1 has a double contribution to the oncogenic process in human malignancies. Firstly, it is a key player in episomal maintenance, making possible the sustainable expression of viral oncogenic products in latently infected cells. Secondly, it provides a direct contribution, due to its interference with various host cell processes, such as cellular gene transcription, protein turn-over, signaling pathways and apoptosis. These mechanisms are described in detail in the next sections of this chapter. One powerful approach to formally demonstrate EBNA1 contributions to the oncogenic process is to suppress its expression or its functions in cellular models of EBV-related malignancies. This has been achieved by numerous investigators, using anti-sense nucleotides, siRNAs or dominant negative EBNA1 mutants. In LCLs and BL cell lines, these treatments have resulted in a partial or complete loss of EBV episomes and a concomitant decrease in cell viability [16,17]. Similar results have been obtained using xenografted BL cells with a recombinant adenovirus to deliver a dominant negative EBNA1 gene [18]. In vitro, one model of artificial NPC cells (NPC-KT) has proven to be sensitive to EBNA1 suppression by RNA-interference [19]. More recently, small molecules with affinity for the DNA binding domain of EBNA1 have also been used to block the replication of the viral genome and reduce the number of EBV genome copies in BL cells [20].

Although EBNA1 is expressed in all forms of EBV-associated malignancy, its expression obeys various modes of regulation depending on the host cells. In most cases, its transcription is driven by the Qp promoter (Bam HI Q fragment promoter) in the context of a type I or type II latency. This applies to EBV-associated BL in situ, Hodgkin’s disease (HD), NPC and EBV-associated gastric carcinomas [21,22,23,24]. In contrast, in the case of EBV-associated DLBCLs (diffuse large B-cell lymphomas with type II or III latency) or post-transplant lymphomas (type III latency), EBNA1 is often transcribed from the Cp promoter or sometimes from both the Cp and Qp promoters [25,26]. As a rule, the EBNA1 protein is detected in the nuclei of most, if not all, malignant cells in tissue sections of EBV-associated malignancies. However, many reports describe cells which are positive for the EBERs (Epstein-Barr encoded RNAs) revealed by in situ hybridization, that are negative for EBNA1 staining [22,27]. This may be due to the poor sensitivity of many antibodies used for EBNA1 detection by immunohistochemistry (IHC) [22,25]. We still have a limited knowledge of the pattern of expression and distribution of the EBNA1 protein in tumour cells in situ. For example, we do not know if expression is more abundant at the level of the invasive front or inside the tumour bed, in the primary or in the metastatic lesions. This is due, at least in part, to the paucity of good antibodies for the detection of EBNA1 in tissue sections. For some time, the 2B4 monoclonal antibody has been regarded as a good reagent for IHC and used in numerous studies [22]. However, 2B4 shows cross-reactivity with cellular proteins, sparking controversies in several previous reports [28,29]. Currently, novel antibodies recommended for IHC are available, but they require validation in a sufficient number of publications. Such reagents will be critical as companion tools for future therapeutic agents aiming to modulate EBNA expression in vivo. Regardless of the host cell, EBNA1 staining displays a characteristic fine granular pattern in interphase nuclei. We do not yet know which type of nuclear structures underlie this speckled distribution. EBNA1 was reported to cause the disruption of PML bodies in epithelial cells, but it is not known to be part of other types of intra-nuclear bodies [30].

The search for EBV subtypes with a possible higher risk of malignancy has been active for several decades. There is currently an acceleration in the field with the advent of high throughput sequencing applied to intra-tumoural EBV isolates [31]. Most authors have focused their attention on polymorphisms of the latent genes *LMP1*, *LMP2* and *EBNA1*. The interpretation of their results is complicated by the unequal geographic distribution of these polymorphisms among healthy carriers. One of the most effective approaches to demonstrate a preferential association is to show a greater frequency of the suspected subtypes in the malignant lesions compared to the viral isolates from saliva and/or peripheral blood of the same individuals. As early as 1996, Bathia et al. reported subtypes of EBNA1 which they often detected in BL tumours, but rarely in peripheral blood isolates [32]. These variants, termed P-thr and V-leu, were defined on the basis of 15 polymorphic codons determining the P or the V category, combined with the sequence of codon 487 (encoding ala, thr, pro or leu). However, these findings were not confirmed by another group who found the same prevalence of the P-thr and V-leu subtypes in peripheral isolates as in the tumour and a strong geographic bias among healthy carriers of BL endemic regions [33]. Subsequently, the V-val was found to be more prevalent in NPC samples than in peripheral isolates from the same donors, at least in South-East Asia [34]. Most of the variant codons thus identified are located in the segment of the *EBNA1* gene encoding the DNA-binding domain of the protein. There is experimental evidence, based on recombinant viruses, that these variations have an impact on the EBV phenotype, with defective suppression of lytic gene expression during the early phase of B-cell infection and impaired ability to transform B-lymphocytes (ibid.). However, there is one report of a possible enhancement of survival after serum withdrawal in epithelial 293 cells expressing the V-val variant; a point that will need confirmation in other cellular backgrounds [35].

It is important to mention the potential of anti-EBNA1 antibodies as a source of biomarkers. Circulating IgG against EBNA1 is consistently detected in healthy EBV carriers. Detection of anti-EBNA1 IgG starts a few weeks after the primo-infection and levels remain at a stable titer throughout life. This doesn’t change through the progress of EBV-associated malignancies, except for NPC patients. Two changes are recorded in these patients: a rise in the concentration of anti-EBNA1 IgG and the *de novo* appearance of anti-EBNA1 IgA. These alterations are concomitant with a rise in IgG antibodies to viral capsid antigen (VCA) and early antigen (EA) and the emergence of the corresponding IgA. Strikingly, these changes often pre-date the development of an invasive tumor. Currently, circulating anti-EBNA1 IgA appears as one of the most potent single markers for the prediction of NPC risk among individuals of endemic regions [36,37]. In the future, circulating anti-EBNA1 antibodies might become interesting biomarkers for early prediction of the tumour response to therapeutic agents designed to trigger a more potent immune response against EBNA1-bearing tumour cells (as discussed below).

### 1.2. EBNA1 Domains and Structure

**Figure 1:**
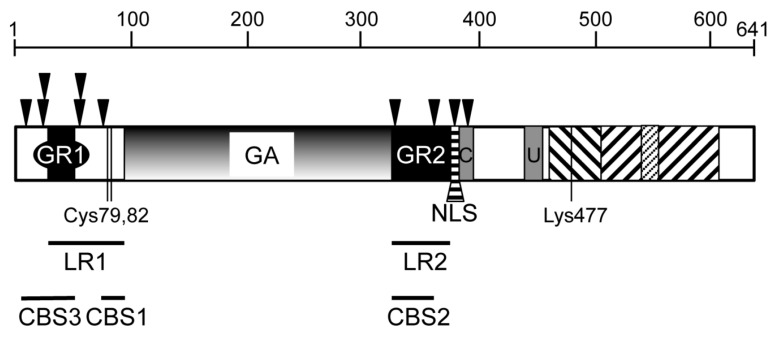
Diagramatic linear representation of the domains of Epstein-Barr virus (EBV) nuclear antigen-1 (EBNA1). The 641 amino acid sequence of the B95-8 strain of EBV EBNA1 is depicted to approximate residue scale (as indicated). GR (black box): Gly/Arg rich domains (GR1:aa33–35, GR2: aa327–377); GA (gradient shading): Gly/Ala repeat (aa90–324); black triangles: predicted phosphorylation sites (Thr8, Thr 20, Ser21, Ser60, Ser62, Ser78, Ser332 Ser365, Ser383, Ser393); NLS (horizontal cross hatched): nuclear localisation signal (378–386); grey boxes (C and U): casein kinase 2 (CK2) and ubiquitin-specific protease 7 (USP7) core binding domains (aa383–395, 436–351 respectively); diagonal cross-hatching: DNA binding and dimerization domain (flanking and core: aa459–503 and 504–607 respectively), with proline-rich loop indicated by fine hatching (aa 538–555); Cys79 and Cys82: key cysteine residues in EBNA1 multimerisation; Lys477: predicted ubiquitination site; LR1 and LR2: linking regions 1 and 2 (aa33–89, aa325–379 respectively); CBS1, 2 and 3: chromatin binding sites 1, 2 and 3 (aa72–84, aa328–365 and aa8–54 respectively).

The primary function of EBNA1 in latent infection is to facilitate viral genome replication once per cell cycle and mediate segregation of the genome into the nuclei of daughter cells. The domains and structure of EBNA1 illuminate this pivotal role in the virus (Figure 1). EBNA1 is a sequence specific DNA binding protein, which acts as a homodimer to cradle the bound viral genome using the C-terminal domain (residues 459 to 607) and thereby carries and attaches the viral genome to the cellular chromatin via the N-terminal and central domains. The crystal structure of the C-terminal domain has been resolved bound to DNA [38,39] and the full protein modelled in silico [40], to reveal that this C-terminal DNA binding domain connects to the remainder of the protein by a string of residues that runs through the major groove of the DNA. In addition, higher order structures have been observed, specifically trimmers of dimers [41]. Several residues in the C-terminal DNA binding domain have been shown to be important for interactions in the dimer [42]. Additionally, contact residues between monomers in the central region of the protein have been identified in the modelled dimer [40]. Within the C-terminal domain is a proline-rich stretch which forms a protruding loop (seen in both the crystal structure and the in silico model), which has been proposed to anchor one monomer to the other in the dimer, with the acidic C-terminal tail of one monomer, part wrapping around the proline loop of the other [40]. The EBNA1 dimers link together when bound to repeats of the DNA recognition sequence, as found in the viral origin of plasmid replication (oriP). This multimerisation is facilitated by two critical cysteine residues (Cys79 and Cys82) [43], which are proposed to coordinate zinc with the same cysteine residues “arm to arm” in the adjacent dimer [40], thus enabling the formation of linked chains of dimers.

Almost one third of the protein is comprised of an imperfect repeat of glycines and alanines (the GAr: residues 90 to 324), with the alanines interspersed by one, two or three glycines (see further below). This domain is highly conserved among different strains of EBV and with few exceptions in the different strains (for example Glu at residues 273 and 274 in the GD1 strain), there are no other residues in this entire stretch. In evolutionarily related EBNA1 sequences of other lymphocryptoviruses (LCVs) infecting different primate hosts, including cynomolgus monkey (*Macaca fascicularis*), rhesus macaque (*Macaca mulatta*) and the baboon genus, the GAr is shorter and interrupted by other residues (notably serine). Interestingly, the GAr is completely absent from the EBNA1 homologue in the new world primate, marmoset LCV [40].

There are two glycine and arginine RGG-rich domains within the protein (termed GR1 and GR2) on either side of the GAr. These are incorporated into regions that have been functionally defined, including regions required for linking EBNA1 to chromatin (in a non-sequence specific manner), termed linking regions (LR1 and LR2: residues 40-89 and 325-379 respectively) [44] and defined by the attachment of EBNA1 to metaphase chromosomes, termed chromosome binding sites (CBS1, 2 and 3: residues 72-84, 328-365 and 8-54 respectively) [45]. Several cellular proteins have been shown to bind to EBNA1 through the GR domains, as described below, and most of these have a demonstrable role in tethering the viral genome to the cellular chromatin. In addition, the GR domains have been shown to have intrinsic DNA binding activity, to AT rich regions, thus termed AT hooks [46].

There are 10 proposed phosphorylation sites in EBNA1 [47]; these are conserved across the different strains of EBV and are located within or around the GR domains. Similarly, several of the arginines in the GR domains are subject to methylation; both phosphorylation and methylation at these sites can regulate EBNA1 function [48].

Between GR2 and the C-terminal DNA binding domain is an intrinsically unstructured region of the protein, demonstrated to bind to at least two cellular proteins, ubiquitin-specific protease 7 (USP7) and casein kinase 2 (CK2) [49,50]. The core binding sites for these proteins are conserved in old world primate LCV EBNA1 homologues, but not in the marmoset LCV homologue [40]. This lead to the hypothesis that binding to these two proteins co-evolved with acquisition of the GAr domain and that therefore, the domains may be functionally co-dependant.

The nuclear localisation signal of the protein (NLS: 379-385, KRPRSPS) lies adjacent to GR2. Sequence analysis predicts a single ubiquitination site within the DNA binding domain, conserved in all EBNA1 LCV homologues at Lys477 [40].

## 2. EBNA1 and Viral Genome Replication and Persistence

In latently infected cells, the EBV genome persists as a multicopy, covalently closed, double-stranded, nuclear episome. When cells proliferate, these episomes replicate once per cell-cycle, during late S phase, via use of the cellular machinery for DNA replication, and are subsequently equally segregated to the daughter cells, such that a constant copy number of EBV genomes is maintained through cell division [15,51,52]. Both replication and segregation depend on the presence of the EBV *cis*-acting oriP and the trans-acting viral protein EBNA1 [13,53].

### 2.1. OriP Structure and EBNA1-Dependent Episomal DNA Replication

**Figure 2:**
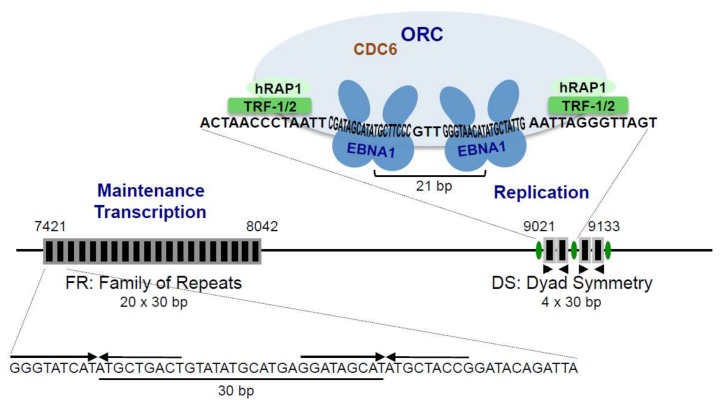
Schematic representation of EBV oriP. The EBV origin of plasmid replication (oriP) is composed of a family of repeats (FR), important for both EBV episome maintenance and EBV gene transcriptional regulation, together with the dyad symmetry (DS), important for viral DNA replication during latency. Individual DNA repeat elements within FR and DS, each carrying an EBNA1 binding motif, are represented by black boxes surrounded by grey outlines. Indicated genome coordinates are from the prototype B95-8 strain. Typical pairs of repeat elements derived from either FR or DS are enlarged to show the nucleotide sequence. EBNA1 recognition sites within FR are represented by head-to-head arrows above the sequence. Regarding DS, two dimers of EBNA1 bound to their DNA recognition sequence are represented (darker blue). The two dimers bind cooperatively to these sites and exact spacing between two EBNA1 binding sites has been found to be critical for origin activity. Binding of EBNA1 dimers to both sites induces a structural distortion of the DNA, probably important for oriP function. Regions of DS adjacent to each pair of EBNA1 recognition sites, carry binding sites for the telomere repeat binding factors, TRF1 and TRF2 (depicted in green) associated with human repressor activity protein 1 (hRAP1) (depicted in lighter green). Binding of these different factors onto the DS element contributes to the recruitment of the cellular origin recognition complex (ORC) (depicted in light blue) together with CDC6 (cell division cycle 6) onto the EBV oriP.

OriP is composed of two functional elements localized 1 kbp apart from one another: the family of repeats (FR) and the dyad symmetry (DS) element [54] (Figure 2). Both elements contain recognition sites for the EBNA1 protein that binds 18 bp palindromic DNA sequences as a homo-dimer [55,56,57,58,59,60]. The DS element, which comprises four high-affinity EBNA1 binding sites arranged in pairs, is required for EBNA1-dependent DNA replication initiation at or close to DS [54,59,61,62]. Within each pair, exact spacing between the two EBNA1-bound dimers appears to be crucial for the DNA replication function [63]. In addition, DS contains three auxiliary repeat elements, located adjacent to each pair of EBNA1-binding sites, that contain motifs resembling telomeric repeats [64] and bind telomere-repeat binding factors, TRF1 and TRF2 [65,66]. The FR element consists of an array of twenty imperfect 30 bp repeats, each containing an EBNA1-binding site followed by a twelve bp AT-rich element [59,67]. In the presence of EBNA1, FR ensures the stable retention of oriP episomes within cells, with a plasmid loss rate of only 3–5% per generation [15,68,69]. FR functions by tethering the viral episomes to human metaphase chromosomes via EBNA1 (detailed below) [70,71,72]. FR also plays a role in viral DNA replication via the stabilization of DS-bound EBNA1, following multiple interactions between FR-bound EBNA1 and DS-bound EBNA1. In addition, FR-dependent tethering of EBNA1 to host chromosomes—especially to perichromatic regions of the host chromatin—during interphase, appears to be essential for the EBV episome to be replicated efficiently [73,74]. Noticeably, even though EBV genome replication during latency takes place in late S phase of the cell cycle, EBNA1 remains constantly associated with both FR and DS elements, throughout the different phases of the cell cycle [75].

The EBNA1 dimerization and DNA-binding domain, which is located in the C-terminal part of the protein (Figure 1), has been crystallized both alone and bound to a DNA fragment containing a specific 18 bp palindromic EBNA1 recognition motif [38,42]. This EBNA1 C-terminal region appears to be organized into two subdomains: a flanking and a core or dimerization domain. The flanking domain makes sequence-specific contacts within the major and minor grooves of the DNA, whereas the core domain—although it is structurally homologous to the complete DNA-binding domain of bovine papilloma virus E2 protein—does not appear to make direct contacts with DNA in the co-crystal structure. It may, however, play a role in sequence-specific DNA-binding, in the context of the assembly of several EBNA1 dimers on multiple sites of the oriP DS element. The structure’s resolution also revealed that EBNA1 binding causes both DNA bending and the appearance of localized regions of helical over- and under-winding. In vivo, EBNA1 has been shown to bind to its recognition sites within the nucleosome and to destabilize its structure by displacing histones from the DNA [76]. In addition to the formation of dimers, EBNA1 can form higher level organized structures resulting from multiple dimer interactions through the LR1 and LR2 domains (Figure 1). Such interactions have been shown to mediate the formation of DNA-looping between the FR and DS elements, which is important for stabilizing EBNA1 interaction at DS [44,77,78,79,80,81,82]. Moreover, it has recently been found that EBNA1 can also form hexameric ring structures (trimers of dimers) via interactions that involve an interface localized within the DNA-binding domain of EBNA1. Destabilization of this higher-ordered structure by the introduction of specific mutations, impact the ability of EBNA1 to cooperatively bind FR and to support viral episome maintenance within the cells [41].

OriP-dependent replication of chromatinized viral episomes is synchronized with host chromosome replication and occurs once in late S phase. It requires the formation of the pre-replication complex (pre-RC) on the DS element. The pre-RC consists of the origin recognition complex (ORC, comprising six proteins called ORC1 to ORC6) associated with cell division cycle 6 (CDC6). With the help of chromatin licensing and DNA replication factor CDT1, this primary complex recruits minichromosome maintenance complex (MCM, composed of MCM components 2 to 7), which is suspected of functioning as a DNA helicase, to form the complete pre-RC (for a review see [83]). In eukaryotes, no specific DNA sequence has been identified for the replication origins or for ORC binding. By contrast, in the case of EBV, the DS element of oriP has been shown to be a specific ORC binding site in the presence of EBNA1, with both ORC and MCM found, by chromatin immunoprecipitation (ChIP), to specifically bind DS [84,85]. The importance of the ORC and Pre-RC in the oriP-dependent replication process, is also supported by the finding that Geminin, an inhibitor of pre-RC formation, inhibits replication from oriP [84]. Although the exact role of EBNA1 in oriP-dependent DNA replication is not yet completely understood, it is reasonable to hypothesize that EBNA1 functions both by changing the structure of DNA and chromatin organisation at the oriP, upon binding its recognition sites, and by recruiting specific elements of the cellular DNA pre-replication machinery (pre-RC) onto the oriP. In this respect, EBNA1 has been shown to interact with the ORC proteins, through the LR1 and LR2 domains [84,86]. In line with this finding, the EBNA1 LR domains responsible for interaction with ORC proteins, can be substituted by several chromatin associated proteins, including high mobility group AT-hook 1 a (HMGA1a), a cellular protein known to specifically target ORC to cellular DNA to define origins of DNA replication [87]. Interestingly, both EBNA1 and HMGA1a have been shown to interact with ORC in an RNA-dependent manner. Since both proteins appear to have affinity for structured G-rich RNA, it has been proposed that G-quadruplex RNA structures could be involved in the HMGA1a- and EBNA1-ORC interactions, although this has not as yet been demonstrated [86]. EBNA1 has also been found to directly interact with purified CDC6, an essential component of the pre-RC which facilitates ORC recruitment, as well as with replication protein A (RPA) and the cellular replicative single-stranded DNA-binding protein [88,89]. In addition, TRF2, which has been found to bind DS cooperatively with EBNA1, also interacts with ORC, thereby probably contributing to the recruitment of the cellular replication machinery [90,91].

### 2.2. EBNA1 Tethering to the Chromosome

During host cell mitosis, efficient segregation of the EBV genomes requires tethering of the newly replicated episomes to human metaphase chromosomes. As mentioned above, this involves EBNA1 bound to the FR region of oriP [46,70,71,72]. EBNA1 can associate with metaphase chromosomes via its N-terminal half, independently of its C-terminal specific DNA-binding domain. The LR domains (delineated above, Figure 1) confer intramolecular “linking” between EBNA1-DNA complexes as revealed by electrophoresis mobility shift assays (EMSA) [78]. The LR1 domain comprises the arginine and glycine rich region GR1 and a unique region (UR1) which incorporates two critical cysteine residues (Cys79 and Cys82), while LR2 essentially demarcates GR2. The GR regions possess intrinsic AT-hook activity allowing binding to AT-rich DNA [46]. The mechanisms responsible for EBNA1 interaction with mitotic chromosomes are still unclear. However, the protein domains involved have been mapped to three independent regions—Chromosome Binding Sites CBS-1, CBS-2 and CBS-3 (Figure 1)—that correlate well with the ability of EBNA1 to confer plasmid maintenance [45,72,92]. The CBS domains include the GR1 and GR2 regions, and it has been proposed that the AT-hook structure within these regions could be directly responsible for EBNA1 attachment to the chromosomes [70,93]. This hypothesis was supported by the observation that HMGA1a, a cellular chromatin-binding protein which associates with chromatin through its AT-hook domain, can functionally replace the amino terminus of EBNA1 both in oriP plasmid replication and partitioning of the viral episome [70,94]. In addition, the use of netropsin—a drug that binds the minor groove of AT-rich DNA—leads to the loss of EBV genomes from epithelial and lymphoid cells, in an AT-hook dependent manner [95]. However, specific deletion of the EBNA1 AT-hook regions does not abrogate EBNA1 targeting to metaphasic chromosomes, suggesting that other mechanisms are likely to be involved [96].

EBNA1 may therefore also interact with chromatin through protein-protein interactions with one or several cellular partners. Human Epstein-Barr binding protein 2 (hEBP2) was the first cellular protein of the sort identified. hEBP2 was isolated from a two-hybrid screen as a putative EBNA1 partner and found to be involved in EBNA1 association with mitotic chromosomes [71,97,98]. hEBP2 binds to the LR2 region of EBNA1 [99], which also corresponds to the CBS-2 region. In a yeast model, hEBP2 was found to be required (in the presence of EBNA1) for the maintenance of a plasmid carrying the EBV FR sequence [100,101]. However, a more recent study, using video microscopy and Förster resonance energy transfer (FRET) microscopy, has demonstrated that although hEBP2 and EBNA1 interact in the nucleoplasm and nucleoli during interphase, they do not interact during mitosis in living mitotic cells [102]. This suggests that the hEBP2/EBNA1 interaction may be indirect or that it plays a role in tethering EBNA1 to the chromatin earlier during interphase.

HMGB2 (high-mobility group box 2), a well-known chromatin component involved in modulating local architecture of the chromatin [103], has also been identified as a partner for EBNA1. Again, by using video microscopy and FRET, EBNA1 interaction with HMGB2 on chromatin was found to occur during both interphase and mitosis, and HMGB2 depletion to partially alter EBNA1 association with the chromosomes. However, HMGB2 depletion did not significantly affect EBV episome maintenance in Raji cells [102]. This suggests that either the interaction with HMGB2 plays a role in a function of EBNA1 other than plasmid maintenance, or that other cellular factors can compensate for HMGB2 absence, in the tethering of EBNA1-bound EBV genomes to mitotic chromosomes.

More recently, regulator of chromosome condensation 1 (RCC1) has been identified as a novel potential mediator of EBNA1 interaction with metaphase chromosomes [96]. RCC1 is the major nuclear guanine nucleotide exchange factor (RanGEF) for the small GTPase Ran enzyme. It is involved in the formation of RanGTP gradients critical for nucleo-cytoplasmic transport [104], mitotic spindle formation, and nuclear envelope reassembly after mitosis [105,106]. RCC1 is a ubiquitously expressed protein that interacts with chromatin via direct contact with both the histones H2A and H2B within the nucleosome and the DNA [107,108]. RCC1 interaction with chromatin is highly dynamic and is stabilized during mitosis [109]. The interaction domains of EBNA1 with RCC1 were found to closely overlap with the CBS regions of the protein known to be important for tethering EBNA1 to the chromosomes [96]. A combination of colocalisation experiments in live cells and FRET analysis revealed that EBNA1 and RCC1 colocalise with the chromatin throughout the cell cycle. However, their proximity varies according to their location within the cell nucleus as well as the phase of the cell cycle: during interphase, although the two proteins appear to be colocalising throughout the cell nucleus, FRET could only be observed at the periphery of the nucleus. The meaning of this interaction at this particular region of the nucleus remains to be determined; during mitosis, FRET was mainly observed in metaphase, indicating a more specific role for the RCC1-EBNA1 interaction at this particular stage of mitosis that precedes segregation of sister chromatids [96]. This observation, together with the overlap between the EBNA1 RCC1-interacting regions and the EBNA1 domains previously characterized for their role in chromosome binding and episome maintenance (Figure 1), argue for an important role for RCC1 in EBV episome tethering to the chromosomes and subsequent episome maintenance. However, this hypothesis will be difficult to prove directly since RCC1 is an essential protein whose downregulation leads to premature chromosome condensation or arrest in the G1 phase of the cell cycle [110].

Efficient EBV DNA-replication and episome maintenance within proliferating cells is clearly dependent on the regulated interaction of EBNA1-bound viral episomes with chromatin throughout the cell cycle. This process likely involves multiple contacts with different chromatin-associated cellular factors orchestrated to play a role at different stages of the cell cycle to both bring EBNA1 to the chromatin and stabilize it once there. Moreover, interaction between EBNA1 and the chromatin could be facilitated by a direct interaction with nucleosomal DNA through the AT-hook domains.

## 3. Regulation of Viral and Cellular Gene Expression by EBNA1

**Figure 3:**
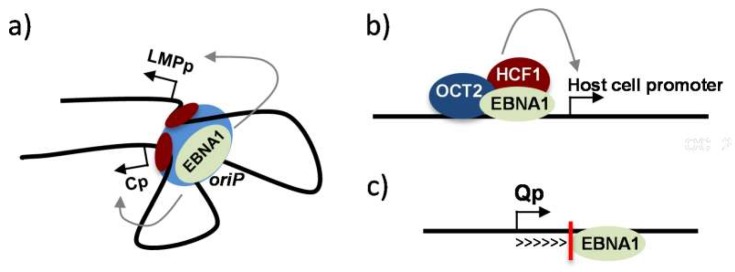
Representation of various mechanisms of gene expression regulated by EBNA1. (**a**) Model showing CCCTC-binding factor (CTCF)/cohesin-mediated DNA looping interactions between oriP and regions close to the Cp and latent membrane protein (LMP) promoters. Such loops bring FR-bound EBNA1 into close proximity with the Cp and LMP promoters, thereby allowing their transcriptional regulation. (**b**) Model of transcriptional regulation by EBNA1 bound onto consensus recognition sites present in the promoters of various cellular target genes. Cooperation with various cellular factors—here OCT2 and HCF1—is likely to be critical for EBNA1-mediated chromatin remodeling and transcriptional regulation outcome. (**c**) By binding to two sites placed in tandem position 10 bp downstream of the transcription start site (TSS) (indicated by the black arrow), EBNA1 represses its own expression from the Qp promoter by a mechanism that blocks processing of the primary transcript.

Besides its role in episome maintenance, EBNA1 binding to the FR element enhances the expression of the viral latency genes responsible for EBV-dependent growth cell transformation, via the transcriptional regulation of the Cp and LMP1 viral promoters located within 15 kbp on either side of oriP [111,112,113,114]. Recent 3C chromosome conformation capture studies indicate that, during latency III, oriP can physically interact with the Cp and LMP promoter regions, with the intervening DNA looping out, thereby bringing EBNA1 in close proximity to the Cp and LMP promoters [115,116] (Figure 3a). Accordingly, EBNA1 positively regulates heterologous promoters when the FR element is placed upstream of these promoters [44,67,113,117]. These data correlate with the observation that the transcriptional activation function of EBNA1 is essential to drive transcription of the EBV transforming genes after infection of primary lymphocytes [118]. Many studies have now revealed that EBNA1 also regulates a wide variety of functionally distinct cellular genes [119,120,121,122,123,124,125] and binds numerous sites within cell chromatin [121,122,124].

The most recent ChIP sequencing (ChIP-seq) analysis, conducted in parallel in four different cell lines with distinct EBV latency types—LCL (type III latency), Mutu I (BL cell line, type I latency), Raji (BL cell line, type III latency with initiation at Wp) and C666-1 (NPC cell line, type II latency)—identified thousands of significant binding sites, most of them being common to the four types of cells [126]. From the analysis of these sites, a consensus recognition motif has been derived that closely resembles those found in the EBV genome, thereby suggesting a direct binding of EBNA1 on many of these sites. Integration of the ChIP-seq data with results of transcriptome sequencing (RNA-seq) identified cellular genes that are likely to be directly regulated by EBNA1 (i.e., genes with promoter-bound EBNA1 and whose expression is modified upon EBNA1 depletion in LCL). These include genes essential for B-cell survival, such as *IL6R*, *KDMC4C*, *EBF1* and *MEF2b*, which are all found to be down-regulated upon EBNA1 depletion. Accordingly, EBNA1 depletion in LCL led to a loss of cell viability with increased apoptosis and reduction of cell proliferation. Moreover, shRNA depletion of *IL6R* and *EBF1* led to important loss of LCL viability. Similarly, shRNA depletion of *MEF2b* resulted in significant loss of cell viability in both LCL and various BL EBV-positive cell lines, but not in EBV-negative B cell lines [126]. In BJAB cells (an EBV-negative BL cell line) constitutively expressing EBNA1, the viral protein has been found to directly up-regulate survivin, an inhibitor of apoptosis expressed in many human cancer cells [125]. Thus, EBNA1 function in transcriptional regulation of host cellular genes could account for the previously reported crucial role of EBNA1 in drastically enhancing B-cell immortalization by EBV [118,127] as well as providing survival signals to EBV-positive BL cells and inhibiting p53-induced apoptosis in non-infected cells [17]. Besides its impact on the expression of genes involved in B-cell survival, EBNA1 directly activates transcription of many other genes involved in various different pathways including several cytokine pathways (IL-18, IL-6, IL-12 and IL-2), the p38 mitogen-activated protein (MAP) kinase network in B-cells [126], the AP1 transcription factor pathway in NPC cells [120] and STAT1 in carcinoma and B-lymphoma cells [119,128]. It can also activate expression of the Gastrokine genes (GKN1 and GKN2)—known for their potential function as tumour suppressors specific for gastric epithelium—in gastric adenocarcinoma (AGS)-EBV infected cells (AGS-EBV) [129]. EBNA1 was also found to have an inhibitory effect on other pathways including cytokine IL-10 and ADIPOQ networks [126].

Until now, the mechanisms by which EBNA1 directly activates transcription are not well understood. A whole genome ChIP-seq approach, to localize EBNA1 binding sites on cellular chromatin, indicated that these sites tend to lie near the transcription start site (TSS) of genes. However, their environment appears to be very diverse since there is no clear chromatin feature (such as specific histone modifications), nor the presence of binding sites for specific transcription factors, that can be associated with the EBNA1 binding sites [126]. Moreover, EBNA1 binding can be associated with either up- or down-regulation of proximal gene expression. Thus, EBNA1 likely cooperates with many different factors to bind DNA and regulate transcription. An example of such a cooperation is found at the EBV oriP, where the cellular factors, host cell factor 1 (HCF1), a component of mixed lineage leukemia (MLL) histone methyltranferase complex, and octamer-binding transcription factor 2 (OCT2), have been found to bind cooperatively with EBNA1 to regulate histone modifications and viral transcription [130] (Figure 3b).

One way in which EBNA1 might favour cellular gene transcription would be by promoting global remodelling of chromatin architecture. In effect, similar to high mobility group A (HMGA) proteins, EBNA1 has been found to promote both chromatin decondensation (without recruiting ATP-dependent remodelers) and the mobilization of linker histone 1. These functions involve the LR1 and LR2 domains of EBNA1, incorporating GR1 and GR2 respectively that resemble the AT-hook motifs of HMGA factors [131]. In addition, EBNA1 could also regulate transcription by contributing to loop formation and promoter selection via its capacity to form homotypic interactions through its linking regions. In this regard, EBNA1 has been shown to promote looping between DS and FR in in vitro experiments [57,77].

Optimal transactivation by EBNA1 requires, besides its DNA-binding domain, the GR1 domain and the adjacent region referred as unique region 1 (UR1: aa65–89) [17,72] (Figure 1). However, while both UR1 and GR1 contributes to transcriptional activation of transfected template, without one of these elements being essential, in the case of a chromosomally integrated template, UR1 is absolutely required [17]. Interestingly, this domain of EBNA1 appears to be specific for EBNA1-mediated transactivation, and dispensable for EBNA1 function in viral episome maintenance [118]. Two cysteine residues within the UR1 domain and the presence of zinc have been found to be crucial for UR1 function by mediating N-terminal multimerization of the protein and cooperative transcription activation [43], and have been modelled to show that the four cysteines present in adjacent dimer “arms” could together coordinate the zinc molecule to link dimers [40]. Several cellular proteins have now been identified that could participate in EBNA1-mediated transcription: bromodomain protein 4 (Brd4), that interacts with acetylated histones to regulate transcription, nucleosome assembly factor 1 (NAP1) and template activator factor I (TAF-I) (via its beta subunit) which belong to the nucleosome assembly family of proteins, and nucleophosmin 1 (NPM1) which has multiple functions including ribosome biogenesis, histone assembly and transcription. These proteins have all been found to interact with EBNA1 in a manner dependent on the presence of the UR1 or GR1 domains, and to participate in EBNA1-mediated transcriptional activation [132,133,134,135].

Although there are many viral and cellular genes that appear to be directly regulated by EBNA1, the protein’s impact extends to the regulation of expression of many other viral and cellular genes by indirect ways. Primarily, by altering various cellular pathways as well as expression of specific transcriptional factors or chromatin regulators, EBNA1 can affect the expression of cellular genes over a wide range. This applies not only to genes transcribed by RNA polymerase II (RNAPII), but also to genes transcribed by RNA polymerase III (RNAPIII). In effect, it has been shown that during EBV infection, EBNA1 protein boosts the production of the general transcription factor complexes, TFIIIB and TFIIIC, required for expression of RNAPIII-dependent genes, including the viral *EBER1* and *EBER2* (Epstein-Barr virus encoded RNA 1 and 2) genes [136,137]. Enhanced expression of RNAPIII-dependent genes, such as those encoding tRNAs and 5S rRNAs, is associated with the transformation of cells and may contribute to the oncogenic environment during DNA tumour virus infection.

In addition, certain more specific mechanisms of gene regulation by EBNA1, have been described. Regarding viral genes, EBNA1 has been found to favour induced-EBV lytic reactivation in epithelial cells, by disrupting promyelocytic leukemia (PML) nuclear bodies [138]. On the other hand, EBNA1 has been shown to inhibit spontaneous EBV reactivation in these same epithelial cells, by a mechanism that involves up-regulation of let-7 microRNA [138,139]. During the B-cell latency III type of infection, EBNA1 represses its own expression from Qp—a viral promoter located 43 kbp downstream of oriP that allows expression of EBNA1 during latency I or II—by a post- or co-transcriptional mechanism that blocks the processing of the primary transcript (Figure 3c). This mechanism involves EBNA1 binding to two sites placed in a tandem position, 10 bp downstream of the TSS, and whose precise location appears to be crucial for the observed effect [140]. Finally, EBNA1 has been found to modify cellular gene expression as a consequence of direct interaction with various proteins. For example, by interacting with ubiquitin-specific protease USP7 (as discussed below), it has been suggested that EBNA1 could interfere with p53’s or MDM2’s own binding to USP7, leading in particular to lower levels of the p53 transcriptional activator, thereby promoting cell survival in response to cellular stress [133,141,142,143,144]. A further indirect mechanism (which will be expanded below), whereby EBNA1 can lead to the induction of the transcription factors E2F transcription factor 1 (E2F1) and c-Myc, could also contribute to a more global change in cellular gene expression.

EBNA1 has been long considered to be essential to the EBV life cycle, because of its role in the replication and maintenance of the viral episome as well as induction of the other EBV transforming genes, but only recently has the full extent of its role in the regulation of cellular gene expression become evident. It is now clear that, together with the other EBV genes expressed during latency, EBNA1 participates in a complete reprogramming of infected cells both to favour their differentiation and ensure the long-term persistence of the virus.

## 4. EBNA1′s Evasion of the Immune System as a New Therapeutic Opportunity against EBV-Related Diseases

Latent viruses show different strategies to evade the host immune system and studies of viral immune evasion have been important to elucidate the pathways that allow the immune system to detect self from-non self. There are numerous and ingenious mechanisms described how viruses thwart the immune system [145,146]. One important target is the presence of non-self antigens on the major histocompatibility (MHC) class I molecules [147]. Peptides presented on MHC class I molecules are recognized by CD8+ T cells and this forms an essential aspect of the self vs non-self selection process and preventing this by one means or another is vital for latent viruses to survive in their host cells. There are various examples of trans-acting viral factors that interfere with different steps in the presentation of viral antigens [148]. For example, herpes simplex viruses (HSV) and human cytomegalovirus (HCMV) each have proteins that target the transport of peptides into the ER where peptides are trimmed and loaded onto MHC class I molecules for further transport to the cell surface. The stability of peptide-MHC class I molecule complexes is dependent on the tapasin chaperone which is a target of HCMV and adenovirus. Another strategy employed by some Herpesviruses is based on proteins that target class I molecules for degradation or internalization. The HIV-1 encoded Nef uses a slightly different strategy that instead prevents the transport of MHC molecules to the cell surface.

EBNA1 can be considered as the Achilles heel of EBV as it is required for viral persistence, but at the same time it is highly antigenic and T cells directed towards EBNA1 epitopes exist in all EBV-infected individuals [149]. Hence, the virus has to allow for EBNA1 to be expressed but must also limit the presentation of EBNA1-derived peptides on MHC class I molecules. As some EBV-carrying cells, like type I BL and memory B cells only express EBNA1 [150], the virus cannot rely on trans-acting mechanisms for immune evasion and EBNA1 instead needs to provide its own way to prevent detection and thence elimination of the host cell [151].

It was initially proposed that EBNA1 could escape antigen presentation by preventing its own proteasomal degradation and thereby avoid EBNA1-derived peptides to be presented to the MHC class I pathway. However, in order to keep a steady state level, the cells will degrade the same amount of a protein that it produces and it is thus, unlikely that any viral strategy based on preventing proteolytic degradation could be successful in starving the class I pathway of peptide substrates. Indeed, it was later shown that EBNA1 harbours a *cis*-acting mechanism whereby it suppresses the translation of its own mRNA and thereby the production of peptide substrates for the MHC class I pathway [152,153]. This model has gained support and has put the focus of EBNA1-mediated immune evasion onto the mRNA translation machinery [154,155]. There are no other similar mechanisms described even though it has been argued that the latency-associated nuclear antigen 1 (LANA1) of Kaposi’s sarcoma-associated virus (KSHV), also a gamma Herpesvirus, might use a similar process [155,156,157]. This self-inhibition of synthesis is mediated by the GAr of EBNA1 and fusion of the GAr to the 5′ of any open reading frame causes suppression of both protein synthesis and antigen presentation. The inhibitory effect of GAr on both EBNA1 synthesis and antigen presentation is length-dependent and a longer domain displays a stronger inhibitory effect [158].

The underlying molecular mechanism of the GAr translation inhibitory action is not clear, nevertheless it was shown that the chaperone Hsp90 is required to suppress translation and also that the GC-rich content of the GAr mRNA plays a role [159,160]. However, changing the 5′-untranslated transcribed region (UTR) of GAr-carrying mRNAs completely abolishes its translation-inhibitory capacity, demonstrating that the ribosome has no difficulty *per se* to read through the GC-rich sequence [158]. This points towards translation initiation as the target mechanism, which is also supported by polysome profiling of GAr-carrying mRNAs [158]. In line with translation initiation being key for GAr-mediated translation control, the U exon in the 5′-UTR of EBNA1 has been shown to contain an internal ribosome entry site (IRES) that regulates the EBNA1 rate of synthesis [161]. 

The GAr sequence needs to be translated to have an effect and fusing it to the 3′-UTR of an mRNA has little effect on translation [153]. Furthermore, changing the codons, while retaining the amino acid sequence, still resulted in a suppression of synthesis [162]. In vitro translation assays using reticulocyte lysates showed that added recombinant EBNA1 does not affect translation, highlighting that it is the translation of the GAr sequence that shuts down its own polysome. Such in vitro assays also revealed that GAr-carrying mRNAs are translated at a similar rate as non-GAr messages at low RNA concentrations, but are suppressed at higher concentrations, suggesting that the GAr targets a positive acting translation initiation factor/s present in limited amount [158].

**Figure 4:**
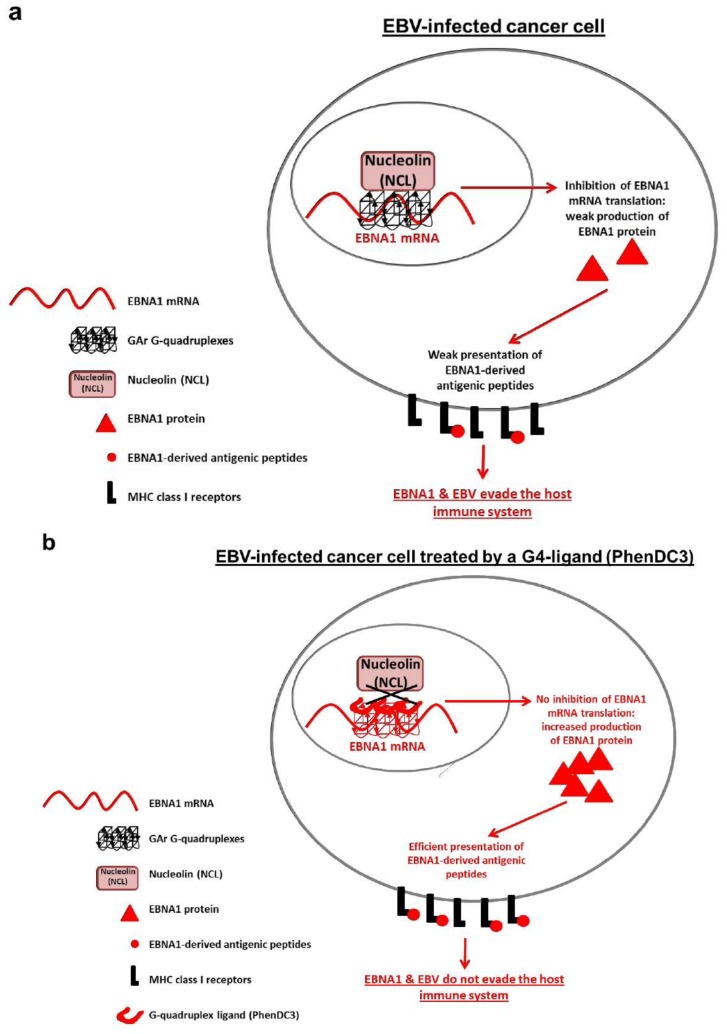
(**a**) Model of the role of nucleolin (NCL) in GAr-based inhibition of translation in cis. NCL binds directly to G-quadruplexes (G4) formed in the GAr-encoding sequence of EBNA1 mRNA, thus inhibiting its translation, thereby leading to weak production of EBNA1 protein and EBNA-derived antigenic peptides, which allow EBNA1 and EBV to evade the immune system. (**b**) Effect of the G4-ligand PhenDC3 on GAr-based inhibition of translation and antigen presentation. PhenDC3 binds to G-quadruplexes (G4) formed in the GAr-encoding sequence of EBNA1 mRNA, thus preventing the binding of NCL, thereby relieving the inhibitory effect of GAr on both translation and antigen presentation. Hence, the NCL-EBNA1 mRNA interaction is a relevant and druggable therapeutic target to unveil tumour cells from EBV-related cancers to the immune system.

Together, these different studies suggest that both the peptide and RNA sequence play roles in controlling translation. A yeast-based genetic assay was developed to model the effect of the GAr on translation in order to identify the cellular factors that mediate GAr-dependent suppression of synthesis in *cis* [163,164,165]. The capacity of GAr to inhibit its own synthesis, including the GAr-length dependency, is conserved in *Saccharomyces cerevisiae*, suggesting that host cell factors involved in the inhibitory effect on translation may also be conserved from yeast to human. Yeast cells lacking the Ade2 protein are red but otherwise healthy, whereas yeast cells expressing a functional level of Ade2 form white colonies. A reporter system expressing a short 43 residue GAr sequence fused to Ade2 was introduced in cells lacking endogenous Ade2. Due to the partial inhibition of translation by 43GAr, this resulted in pink colonies expressing an intermediate level of 43GAr-Ade2 [165]. The introduction of a yeast genomic overexpression library identified the multifunctional RNA binding protein nucleolin Nsr1 as mediating GAr-dependent translation control [157]. Overexpression of *NSR1,* or of its human homologue nucleolin (NCL), exacerbates GAr-dependent translation inhibition *in cis* in yeast and mammalian cells whereas knockout of *NSR1* in yeast or downregulation of NCL in mammalian cells suppresses it. NCL is also required for suppression of EBNA1 antigen presentation [157]. The GAr-encoding mRNA sequence forms G-quadruplex structures (G4) [155] and NCL directly binds these non-canonical structures (Figure 4a and [157,166,167]). By competing for the G4-NCL interaction, using the G4 ligand PhenDC3, the rate of EBNA1 synthesis and the presentation of antigenic peptides is enhanced (Figure 4b and [157]). Importantly, pyridostatin (PDS), another G4-binding compound, which was shown to further suppress EBNA1 synthesis in an in vitro transcription-translation coupled assay [155] had no effect on EBNA1 expression *in cellulo* and did not compete for the binding of NCL [157]. This discrepancy may be explained by the weaker affinity of PDS for the G4s of the GAr-encoding sequence of EBNA1 mRNA [157]. Alternatively, the mode of binding of PDS to G4s may be different compared to PhenDC3 and thereby unable to prevent the binding of NCL. These studies have greatly helped to explain the translation inhibitory activity of the GAr, but what about its capacity to prevent proteosomal degradation of proteins to which it is fused? This was initially thought to explain the *cis*-acting mechanism whereby EBNA1 evades the immune system and was based on the fact that fusing the GAr to the IkBα protein prevents GAr-IkBα degradation via the 26S proteosomal pathway [168]. However, more detailed studies showed that the position of the GAr within the fusion protein significantly alters its effect on proteasome mediated degradation [169]. For example, p53 is targeted for degradation via the 26S proteasome pathway by the E3 ubiquitin ligase MDM2 and fusing the GAr to the N-terminus of p53 resulted in a dramatic increase in the MDM2-dependent rate of degradation, whereas fusing it to the C-terminus instead caused stabilization and at the same time resulted in an MDM2- and proteasome-dependent partial degradation. The location-dependent effect of the GAr could be observed on several substrates, suggesting a model in which the GAr affects the unfolding of the ubiquitinated substrate at the 19S subunit of the 26S proteasome [169,170]. It is an interesting possibility that the effect of the GAr peptide on chaperone-dependent protein unfolding mirrors the effect of the encoded GAr peptide on translation. This notion is supported by the observation that inserting a small polar residue in every eight codon renders the GAr non-functional for translation suppression [158].

## 5. EBNA1′s Oncogenic Effects and Partners

### 5.1. C-Myc, E2F1, PI3K and BL

C-Myc is a basic, helix-loop-helix/leucine zipper transcription factor. It heterodimerises with Max, to bind to its target recognition sequence (E-box with the consensus 5′-CACGTG-3′). Through this binding, c-Myc containing complexes can either activate or repress a plethora of genes [171,172], with 4296 potential c-Myc binding sites identified in an EBV-immortalised human B-cell line [173] and thousands of genes found to be Myc-responsive in many systems [174]. C-Myc plays a central role in promoting cell proliferation and cell growth, regulating genes involved in cell cycle, metabolism, apoptosis and protein biosynthesis, including ribosomal biogenesis [175,176,177]. Consequently, c-Myc expression is tightly regulated at all levels: transcription (both initiation and elongation), mRNA stability and translation and protein turnover (reviewed in depth in [174]).

The neoplastic B-cells of BL, both EBV associated and EBV negative, harbour a characteristic translocation involving the *c-Myc* locus (on chromosome 8) with one of the immunoglobulin (Ig) loci (IgH on chromosome 14, or less frequently Igκ or Igλ on chromosomes 2 and 22 respectively) [178]. Interestingly the break points at the *c-Myc* locus differ between endemic BL (eBL, which is >95% EBV positive) and sporadic BL (approximately 15% EBV positive), which could reflect a viral influence in the process or selection. Typically, the breakpoints at the *c-Myc* locus in sporadic BL occur within the immediate 5′ region of the gene, while in eBL they are usually more distal [179]. The tumours show high expression of c-Myc and are largely Ki-67 positive (greater than 95% of the neoplastic cells), indicative of a high proliferative index (reviewed in [180]). Notably, the translocation that occurs in BL is responsible for a shift in the *c-Myc* promoter usage ratio, between P1 (directly 5′ to exon 1) and P2 (within exon 1). In normal cells, transcripts are predominantly derived from P2, while in BL the PI/P2 usage ratio changes to between 1:1 to 4:1, or if both promoters are disrupted by the mutation, to the minor P3 promoter within intron 1; all leading to a loss of the regulating block to transcription elongation [174,181]. Additionally, likely due to the proximity of the immunoglobulin loci, the translocated *c-Myc* allele in BL is subject to somatic hypermutation [182] and mutations that affect the phosphorylation sites ser62 and thr58 can influence protein stability.

Recently, a direct link between EBNA1 and c-Myc expression was revealed with the identification of a unique mechanism mediated by the GAr of EBNA1 [183]. The mRNA translation suppression *in cis* by the GAr (see Section 4) causes a translational stress that activates E2F1 expression via phosphoinositide-3-kinase-δ (PI3Kδ) dependent signalling. This increase in E2F1 activity causes the induction of c-Myc. The class 1 PI3Ks are heterodimeric enzymes comprised of a p110 catalytic subunit (α, β, γ or δ) and a regulatory subunit (p85/55/50-α, p85-β, p55-γ, p101, p84). While p110α and p110β are expressed ubiquitously, the p110δ catalytic subunit is primarily expressed in leukocytes but can also be detected in some tumour cells of epithelial origin [184]. Specific inhibition of PI3Kδ using chemical inhibitors, or knock out using siRNAs, abrogates EBNA1-induced E2F1 and c-Myc activation [183]. The GAr is dispensable for EBNA1’s known biochemical properties and, as it suppresses EBNA1 expression and has a strong tendency to cause aggregates, it is most often deleted in EBNA1 studies, which might help to explain why this has not been observed previously.

E2F proteins are transcription factors that regulate mitosis and genomic integrity and their transcriptional activity is modulated by binding to the cell cycle inhibitory pocket proteins pRb, p107 and p130. As such, the E2F-Rb pathway is frequently deregulated in cancer. E2F transcriptional activators (E2F1, E2F2 and E2F3) induce the expression of multiple cell cycle genes, as well as *c-Myc* [185]. E2F1 and c-Myc operate in a feed forward loop, each acting upon the promoter of the other. Interestingly, the E2F binding site at the *c-Myc* locus lies between P1 and P2 and is not thought to influence P1 [186]. However, evidence suggests that E2F1 in complex with pocket proteins (pRb, p107, p130) and histone deacetylases (HDAC) inhibit transcriptional elongation from this site [186]. Conversely, release of E2F1 from the protein complex (by phosphorylation of the pocket proteins or excess E2F1), may stimulate the processive transcriptional elongation from P2 [186,187]. In accord with this, inhibition of PI3Kδ affects c-Myc expression in the B95.8 LCL and in EµEBNA1 transgenic mouse lymphoma cells in which the *c-Myc* gene is intact, but not in Raji BL cells carrying a translocated *c-Myc* gene [183]. The EµEBNA1 mice harbour a transgene incorporating the full length, unmodified EBNA1 (of the B95.8 strain) linked to the immunoglobulin heavy chain intronic enhancer (designated Eµ) to direct expression to B-cells [188].

A further link in the chain is suggested by the observation that IL-2 promotes the survival of EµEBNA1 transgenic B-cells, both before tumour development and in the subsequent lymphomas, consistent with the increased expression of the IL-2 receptor-α chain CD25 at the surface of the transgenic B-cells [128,189,190]. EBNA1-induced expression of CD25 was also observed in an HL cell line [191]. IL-2 has been shown to stimulate *c-Myc* transcription through an upstream STAT4 binding site [192] and through the PI3K pathway [193,194]. Additionally, IL-2 induces activation of E2F via the PI3K pathway [195] and PI3K signalling can inhibit the degradation of c-Myc through regulating GSK3β phosphorylation of c-Myc, thereby promoting its stability [196]. The relevance of the PI3K pathway in EBV-related disease is further suggested by the fact that mutations in *PIK3R1*, the gene that encodes the alpha regulatory subunits (p85/55/50-α) of the PI3K enzyme, are common in BL [197]. In addition, PI3K signalling and c-Myc have been identified as cooperative factors in modelling the development of BL in mice [198]. Aside from EBNA1, other viral genes can also activate the PI3K pathway, specifically EBNA2, LMP1 and LMP2A [199,200,201,202,203] and so in cells where all latent EBV antigens are expressed, there could be functional synergy, or even overlap, in activating PI3K. With respect to EBV infection, it may be germane that patients who suffer from activated PI3Kδ syndrome (APDS) caused by mutations in either the PI3K encoding gene (*PIK3CD*) or in *PIK3R1*, are immunosuppressed and tend to suffer from chronic herpesvirus viraemia including EBV and have an increased risk of developing B-cell lymphoma [204].

### 5.2. The USP7, p53, MDM2 Connection

**Figure 5:**
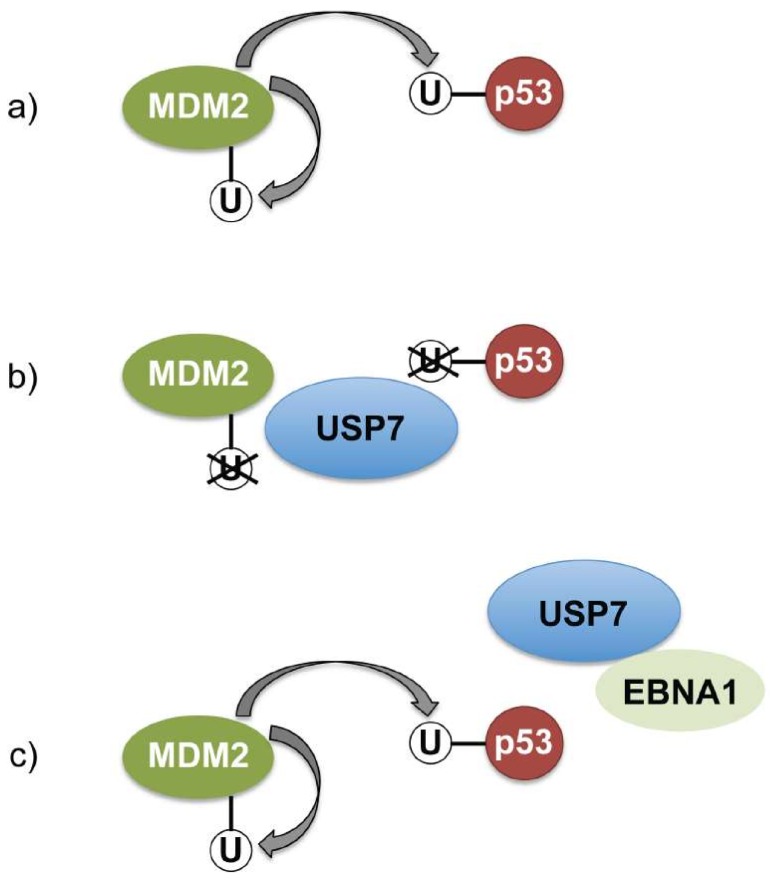
(**a**) MDM2 acts to ubiquitinate both p53 and itself, which will lead to the degradation of the proteins, thus maintaining low levels despite continued expression. (**b**) Under conditions of stress, USP7 functions to remove the ubiquitin groups and thus, protein stabilization. (**c**) The interaction between USP7 and cellular or viral factors provides a potential mechanism to regulate de-ubiquitination of MDM and p53.

EBNA1 associates with the ubiquitin specific protease-7 (USP7 aka HAUSP) through a binding motif in the former located just N-terminal to the DNA binding domain (Figure 1) [133]. USP7 also binds the tumour suppressor protein p53 and the ubiquitin ligase MDM2 (amongst other proteins, including MDMX and FoxO4A) and in so doing, removes ubiquitin moieties that would otherwise target these substrate proteins for degradation by the proteasome (Figure 5). Thus USP7 is able to stabilise both p53 and MDM2 [205,206,207] and it has been hypothesised that EBNA1 can compete for the binding of USP7 to MDM2 and thereby affect the p53 pathway [133,141,143].

Several isoforms of MDM2 are expressed in BL cell lines (both EBV positive and negative), additionally high levels of MDM2, along with high levels of c-Myc and E2F1 are consistently detected in EµEBNA1 transgenic mouse lymphomas [128]. Indeed, the EµEBNA1 transgenic tumour cell viability is dependent upon MDM2, demonstrated by MDM2 inhibition, suggesting a direct role for the activation of MDM2 in the EBNA1 mediated tumourigenic process [128]. The MDM2 story is complex and is not reviewed in depth here. Not only is the mutual regulation between p53 and MDM2 multi-layered, acting at several levels: transcriptional control, translation control, post translational modification (reviewed in [208]), but the *MDM2* gene is multiply spliced and can express numerous protein isoforms, each retaining only part of the functionality of the full protein (reviewed in [209]). Interestingly, the observation that inhibition of MDM2 in EµEBNA1 transgenic lymphoma cells led to reduced EBNA1 expression alongside that of E2F1, suggests a further link between EBNA1 and E2F1 [128].

## 6. EBNA1 as Therapeutic Target

EBNA1 has no cellular homologue and thus, represents an interesting therapeutic drug target that distinguishes EBV-carrying tumour cells from normal tissue. EBNA1 is essential for viral genome replication and propagation and drugs that target EBNA1 and suppress its activity will cripple the virus and thereby any oncogenic activity not only linked to EBNA1 but also to the virus. The observation that EBNA1’s induction of E2F1 requires PI3Kδ suggests that PI3Kδ inhibitory compounds such as idelalisib, might be used to prevent EBNA1-mediated oncogenic stimuli. There are multiple routes by which EBNA1 inhibitory drugs may be developed and there are several compounds already in existence. The DNA binding activity of EBNA1 can be disrupted [20,210], homodimerisation of EBNA1 can be inhibited [211]. Blocking the interactions between EBNA1 and cellular proteins, e.g., USP7, CK2 or proteins which associate through the GR domains including RCC1 or targeting its oncogenic partners, such as MDM2, offers alternative routes [128]. Finally, disruption of EBNA1’s immune evasion mechanisms, by manipulating the GAr translation suppression activity [157,163,164,165], may render EBV-carrying cancers targets for the immune system. Moreover, preclinical models are available to test candidate therapeutic drugs in vivo [188,212,213]. It has been over 50 years since the discovery of EBV and now, finally, we might have started on a path towards developing drugs that specifically target EBV-related diseases.

## 7. Perspectives

Aside from the recently reported role of NCL in the EBNA1 immune evasion mechanism, NCL is also reported to be involved in EBNA1-dependent EBV episome maintenance and transcription through binding to the N-terminal region of the EBNA1 protein [214]. Thus, NCL regulates EBNA1-dependent EBV episome maintenance and transcription as well as self-limitation of EBNA1 expression and it will be interesting to see if there is a connection between these two events, both from a physiological and a mechanistic point of view. For example, low NCL levels could compromise genome maintenance and transcription but, as it also stimulates EBNA1 synthesis, could thereby provide a potential feedback system to ensure that an optimal level of EBNA1 is maintained. Conversely, at high NCL concentrations, genome maintenance and transcription would be efficient, but EBNA1 mRNA translation self-limitation might be exacerbated, thereby limiting EBNA1-derived antigen presentation. Accordingly, it will also be interesting to see if the binding of NCL to the EBNA1 mRNA plays a role in its association with the EBNA1 protein. Finally, like NCL, the EBNA1 protein has also been shown to bind G4s [215]. Hence an appealing possibility would be that, in addition to NCL, EBNA1 also participates in GAr-dependent self-limitation of EBNA1 mRNA translation. Of note, this hypothesis would provide a basis for the role of the EBNA1 protein in the limitation *in cis* of its own mRNA translation.

Data revealing that PhenDC3 can enhance EBNA1 synthesis demonstrate that the immune evasive effect of the GAr is druggable and that different G4-binding compounds do not have the same effect on the mRNA-NCL interaction, most probably depending on their relative affinity for G4s of EBNA1 mRNA and/or of their mode of binding to G4s [157]. By interfering with the interaction between NCL and the G4s of EBNA1 mRNA, thereby increasing the rate of EBNA1 mRNA translation, it is thus possible that one can render EBV-carrying cancer cells targets for the patients’ own immune system. Hence, the NCL-EBNA1 mRNA interaction may represent a bona fide and original therapeutic target to treat EBV-related cancers [157]. This hypothesis is currently being explored.

With respect to the interaction between EBNA1 and USP7, it is not yet clear exactly how this impacts the regulatory loop that exists between MDM2 and p53. MDM2 catalyses the ubiquitination of both p53 and itself, while USP7 can remove the ubiquitin groups from both proteins [205,206,207]. The levels of USP7 appear to be critical in determining whether p53 or MDM2 is preferentially stabilised; with moderate reduction of USP7, p53 is destabilised, while loss of USP7 results in MDM2 loss and p53 stability [216]. If the EBNA1-USP7 association competes with binding to these cellular proteins, EBNA1 may effectively sequester USP7 [133,141,143]. Therefore the balance between USP7 and EBNA1 levels would be predicted to have different outcomes, such that low level EBNA1 resulting in slightly reduced available USP7 would favour MDM2 stability and p53 degradation and cell survival; conversely high levels of EBNA1 would result in proportionally less available USP7, thus might favour p53 stability and cell cycle arrest. This could go some way to explain why high levels of EBNA1 can result in growth arrest in some cells [217], and also provides added rational behind the importance of controlling the level of EBNA1 expression in EBV latently infected cells (as mediated through the GAr of EBNA1 and NCL, described above).

With the recent indication that MDM2 plays a role in EBNA1-mediated tumorigenesis and that inhibition of MDM2 in EµEBNA1 transgenic lymphoma cells leads to reduced EBNA1 expression alongside that of E2F1 [128], it is interesting to speculate about the role of MDM2 in this process. The MDM2-p53 pathway is implicated in responses to ribosomal stress and the central region of MDM2 has been shown to bind to 16 ribosomal proteins in responding to stress [218]. Furthermore, MDM2 interacts with, and by preventing ubiquitination, can prolong the half-life of E2F1 [219], while in a negative feed-back loop, E2F1 inhibits MDM2 expression in p53 wild type cells [220]. The observation that p53 is commonly mutated in BL [197,221] would release this negative feedback loop. Thus the association of EBNA1 with USP7 may be focused as much on MDM2 functionality as on p53. This lends support to the idea that the GAr of EBNA1 and the USP7 and CK2 binding sites may have co-evolved (with all three domains absent from the marmoset lymphocryptovirus EBNA1 homologue) [40], although the role of CK2 binding by EBNA1 is yet to be understood. Acquisition of the GAr provides the virus with a mechanism to limit EBNA1 expression (and thus immune detection) and tie EBNA1 expression to the cell cycle, while UPS7 binding could interfere with the subsequent ribosomal stress response.

Studies of viral proteins have provided windows of opportunities to understand many aspects of cell biology. Accordingly, understanding the mechanisms of what cellular pathway the GAr exploits to target its own synthesis will continue generating more exciting data on *cis*-mediated regulation of mRNA translation and will shed light on why and how EBNA1 is using both its mRNA and peptide sequence to control its own rate of synthesis and how this relates to the production of peptides for the MHC class I pathway.
